# Identification of a fall armyworm (*Spodoptera frugiperda*)-specific gene and development of a rapid and sensitive loop-mediated isothermal amplification assay

**DOI:** 10.1038/s41598-022-04871-2

**Published:** 2022-01-18

**Authors:** Angelina F. Osabutey, Bo Yoon Seo, A-Young Kim, Thu Anh Thi Ha, JinKyo Jung, Georg Goergen, Ebenezer Oduro Owusu, Gwan-Seok Lee, Young Ho Koh

**Affiliations:** 1 Department of Biomedical Gerontology, Hallym University Graduate School, Chuncheon, Gangwon-Do Republic of Korea; 2 Ilsong Institute of Life Sciences, Hallym University, Yeongdeungpo-gu, Seoul, Republic of Korea; 3 Crop Protection Division, National Institute of Agricultural Science, Rural Development Administration, Wanju, Jeollabuk-Do Republic of Korea; 4 Crop Cultivation and Environment Research Division, National Institute of Crop Science, Suwon, Gyeonggi-do Republic of Korea; 5 IITA Biological Control, Center for Africa, Tri Postal, 08BP 0932 Cotonou, Benin; 6 Department of Animal Biology and Conservation Science, University of Ghana, Legon-Accra, Ghana

**Keywords:** Entomology, Agricultural genetics

## Abstract

The fall armyworm [FAW, *Spodoptera frugiperda* (J E Smith)], a moth native to America, has spread throughout the world since it was first discovered in Africa in 2016. The FAW is a polyphagous migratory pest that can travel over long distances using seasonal winds or typhoons because of its excellent flying ability, causing serious damage to many crops. For effective FAW control, accurate species identification is essential at the beginning of the invasion. In this study, the FAW-specific gene Sf00067 was discovered by performing bioinformatics to develop a fast and accurate tool for the species-specific diagnosis of this pest. An Sf00067 loop-mediated isothermal amplification (LAMP) assay was developed, and optimal conditions were established. The Sf00067 6 primer LAMP (Sf6p-LAMP) assay established in this study was able to diagnose various genotype-based strains of FAW captured in Korea and FAWs collected from Benin, Africa. Our FAW diagnostic protocol can be completed within 30 min, from the process of extracting genomic DNA from an egg or a 1st instar larva to species determination.

## Introduction

The fall armyworm [FAW, *Spodoptera frugiperda* (J E Smith)], an indigenous moth in tropical and subtropical regions in the Americas, is distributed between La Pampa, Argentina and southern Florida and Texas, USA and occurs throughout the year^1,2^. FAW is a polyphagous moth reported to feed on at least 353 plants, including the economically important crops maize, rice, wheat, sorghum, sugarcane, beet, cotton, tomato, potato, and pasture grasses^3^. Since FAW cannot undergo diapause during winter, it can only survive where the average winter temperature is above 10 °C (50 °F)^4^. Thus, it was reported that FAW only survives in southern Florida and Texas in North America during winter. However, the great migration ability and fertility of FAW makes its occurrence possible during summer in Ontario and Quebec, Canada, which are 2000 km away from southern Florida and Texas^1^.

The first invasion of FAW to other continents was confirmed by an outbreak of FAW in South West Nigeria and Ghana, Africa, in January 2016. Within 2 years, FAW rapidly spread to all countries in sub-Saharan Africa^2,5^. Currently, FAW is not found in North Africa, but climate conditions in some granaries of Morocco, Sudan, and Egypt are suitable for the breeding of FAW^1,2^.

In Asia, after being discovered on the Indian continent in 2018, FAW was discovered in Bangladesh, Thailand, Myanmar, Sri Lanka, etc.^6^. After the first report from Yunnan Province, China, at the end of 2018, FAW occurred all over southern China, including Guangdong Province, Fujian Province, and Zhejiang Province^7^, which is believed to be one of the origins of the migratory insects that flew to Korea. In Korea, FAW was first discovered on Jeju Island approximately six months later than in China and was captured at the corn cultivation complex in Yeonggwang, Jeollanam-do, at the end of July^8^. The damage caused by these migratory insect pests is minimal at the beginning of invasion due to small number of adults, but a female adult can spawn more than 1000 eggs. The egg development period of FAW is approximately 2–4 days, and the larval period is approximately 14–22 days^2^. Thus, damage to crops caused by FAW will be rapidly increased within 1 or 2 weeks after the arrival of adults. Unlike insect pests that can be removed during quarantine processes, migratory insect pests are very likely to invade the crop cultivation area of Jeju and the southwest coast of Korea at any time because their flight to Korea may be aided by the seasonal winds blowing from Southeast Asian countries to Korea. Thus, to reduce the damage caused by FAW, accurate and rapid identification of this pest is necessary at the beginning of an invasion.

The migration and occurrence of FAW can be monitored by sex pheromone traps^8^. Nevertheless, a number of other moths are simultaneously captured in FAW pheromone traps, and the morphology of the moths could be damaged in the process of moths becoming secured to the commonly used sticky traps. Thus, the classification of trapped moths based on their morphology is, at times, difficult. Furthermore, there is no significant difference among the morphologies of eggs or larvae of moths, and the identification of FAW can also be accomplished by molecular tools based on cytochrome oxidase I (COI) DNA barcodes^8,9^. However, because species identification using COI DNA barcodes requires sophisticated molecular biological instruments to perform PCR and DNA sequencing, these methods are not suitable for on-site diagnosis.

In this study, rapid and accurate on-site FAW identification tools were developed using loop-mediated isothermal amplification (LAMP). LAMP is a method of amplifying a target gene using its 4 to 6 specific primers at isothermal temperature using a LAMP polymerase. Because LAMP has high sensitivity and accuracy, it is currently being used in various molecular diagnostics^10–12^. Our LAMP-based FAW diagnostic protocol can be completed within 30 min, from the process of extracting genomic DNA from one egg or a 1st instar larva to species determination.

## Results

### The FAW specificity of Sf00067 was confirmed by conventional PCR

**Figure 1:**
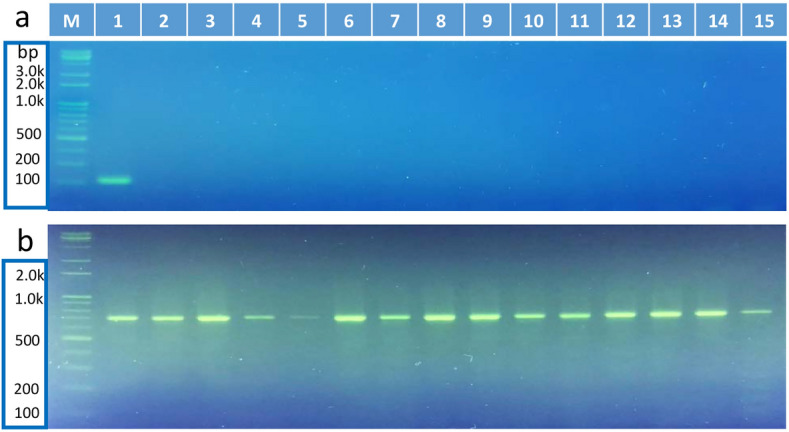
Sf00067 was a FAW specific biomarker. (**a**) When the Sf00067 F3 and B3 pair was used to perform PCR with genomic DNA from 15 moth species, only a 209 bp DNA band was detected from FAW genomic DNA. (**b**) LCO1490 and HCO2198 primers were used as loading controls. Moth species used in this study were as follows: 1. FAW, 2*. Axylia putris*, 3*. Mythimna loreyi*, 4. *Discestra trifolii*, 5. *Hermonassa cecilia*, 6.* Anomis flava*, 7. *Aedia leucomelas*, 8.* Pleuroptya ruralis*, 9.* Mythimna separata*, 10. *Lacanobia contigua*, 11. *Helicoverpa armigera*, 12. *Mamestra brassicae*, 13*. Spodoptera exigua*, 14. *Spodoptera litura*, 15. *Plutella xylostella.*

To verify that Sf00067 was a FAW-specific biomarker, conventional PCR was performed with genomic DNA extracted from 15 moth species (Fig. 1). Among the 15 moths tested, 10 species were collected from FAW pheromone traps (No. 1–10), and 5 moth species were raised in the insectarium (No. 11–15). When the Sf00067 Forward primer 3 (F3) and Backward primer 3 (B3) pair was used for PCR, a 209 bp DNA fragment was only detected from FAW genomic DNA but not from the genomic DNA from the 14 other moth species. In contrast, when the universal COI primer pair was used for PCR, approximately 700 bp DNA fragments were detected from all samples (Fig. 1b), suggesting that genomic DNAs from other moths were good enough for performing PCRs. Taken together, this result suggested that Sf00067 is a FAW-specific biomarker.

### Incubation for 35 min at 65 °C was optimal for the Sf00067 4 primer-LAMP assay

**Figure 2:**
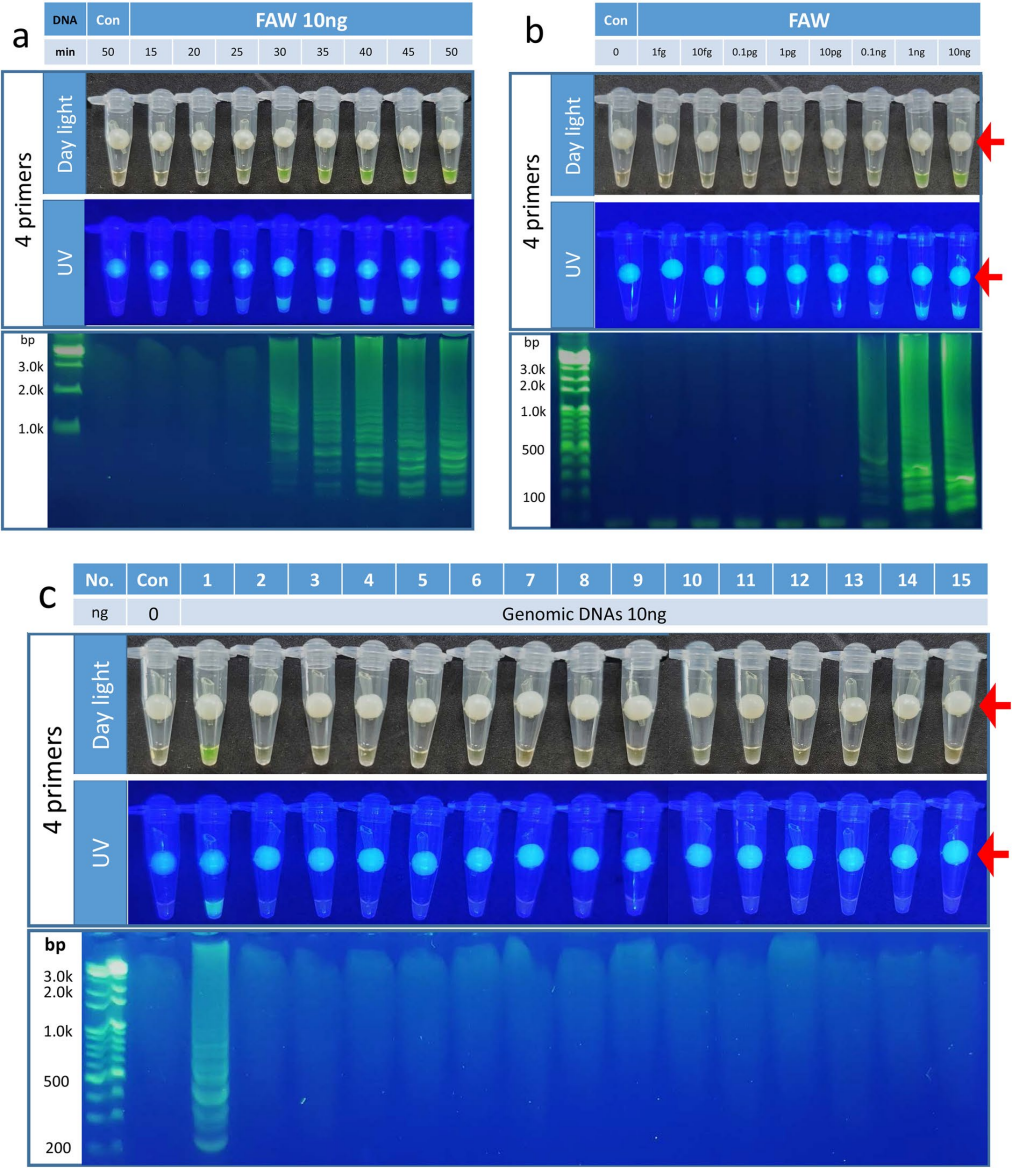
The optimum incubation periods, detection limits and FAW species specificities for the Sf4p LAMP assay. (**a**) After 35 min incubation, the fluorescence signals and the patterns of ladder-shaped DNA fragments were similar among Sf4p LAMP assays. No amplification was detected after 50 min of incubation when genomic DNA was not added (Control = Con). (**b**) When serially diluted FAW genomic DNAs were used to determine detection limits for the Sf40 LAMP assay, fluorescence signals and DNA amplification were detected above 0.1 ng/µl of FWA genomic DNA. The fluorescence intensities and patterns of DNA fragments were similar above 1 ng/µl of FAW genomic DNA. (**c**) When genomic DNAs of moths captured by FAW sex-pheromone traps (1–10) or raised in the insectarium (11–15) were used for the Sf4p LAMP assay, strong fluorescence signals and amplified DNA fragments were observed from FAW genomic DNA. Moth species used in this study were as follows: 1. FAW, 2. *Axylia putris*, 3. *Mythimna loreyi,* 4. *Discestra trifolii*, 5. *Hermonassa cecilia*, 6. *Anomis flava*, 7. *Aedia leucomelas*, 8. *Pleuroptya ruralis*, 9. *Mythimna separata*, 10. *Lacanobia contigua*, 11. *Helicoverpa armigera,* 12. *Mamestra brassicae*, 13. *Spodoptera exigua*, 14. *Spodoptera litura*, 15. *Plutella xylostella*. The red arrows indicate the contamination-free SYBR green delivery device.

When Sf00067 4 primer (Sf4p)-LAMP assays were performed with 10 ng of FAW genomic DNA, strong fluorescence signals from tubes were detected under day light and UV light after 30 min incubation at 65 °C. The fluorescence intensities and patterns of ladder-shaped DNA fragments that are typical of LAMP assays were similar after 35 min of incubation (Fig. 2a). This result suggested that the optimum incubation period for the Sf4p-LAMP assay was 35 min.

### The detection limit of Sf4p-LAMP assay was 0.1 ng/μl

To determine the detection limits of Sf4p-LAMP assay, serially diluted FAW genomic DNAs were used. Fluorescence signals and DNA amplifications started to be detected from 0.1 ng/μl (Fig. 2b). The intensities of fluorescence signals and patterns of DNA fragments were similar when more than 1.0 ng/μl of FWA genomic DNA was used. Thus, the detection limit for the Sf4p-LAMP assay was 0.1 ng/μl.

### Sf4p-LAMP assay was specific to FAW genomic DNA

To investigate the specificities of the Sf4p-LAMP assay, genomic DNAs of 15 moths, including 10 species of moths captured in FAW sex-pheromone traps and 5 related moths raised in the insectarium, were examined by the Sf4p-LAMP assays (Fig. 2c). When Sf4p-LAMP reactions containing 1.0 ng/μl of genomic DNA from 15 month were incubated for 50 min at 65 °C, strong fluorescence signals and amplified DNA fragments were detected from FAW genomic DNA only.

### Improving the efficiency of the Sf4p-LAMP assay by adding loop primers

It has been shown that the efficiency of the LAMP assay could be enhanced by adding one or two loop primers^13,14^. When the Sf00067 loop forward primer (SfLF)-LAMP assay was performed, fluorescence signals and DNA amplifications were detected after 20 min. After 25 min of incubation, the fluorescence signals and patterns of DNA fragments were similar (Supplementary Fig. 1a). These results suggested that the addition of the loop forward primer (LF) to the Sf4p-LAMP assay mixture reduced the incubation period by 10 min compared with that of the Sf4p-LAMP assay mixture. In addition, the detection limit of the SfLF-LAMP assay was significantly enhanced compared to that of the Sf4p-LAMP assay, as the fluorescence signals and DNA amplifications were found in assays containing 10 pg/μl or more FAW genomic DNA (Supplementary Fig. 1b). Next, a SF00067 loop reverse primer (SfLB)-LAMP assay containing the loop backward primer (LB) and 4 primers was performed to examine any changes in fluorescence signals and patterns of amplified DNAs (Supplementary Fig. 1c). In contrast to the SfLF-LAMP assay, the incubation period of the SfLB-LAMP assay was similar to that of the Sf4p-LAMP assay. Fluorescence signals and DNA amplifications were detected after 30 min. There was no significant difference in fluorescence signals or patterns of DNA amplification among SfLB-LAMP assays after 35 min. These results suggested that adding LB did not reduce the incubation period. However, the detection limit of the SfBF-LAMP assay was significantly enhanced. Strong fluorescence signals and large amounts of amplified DNA were detected when more than 10 pg of FAW genomic DNA was used (Supplementary Fig. 1d).

### The Sf00067 6 primer-LAMP (Sf6p-LAMP) assay reduced the reaction duration and increased the sensitivity to FAW

**Figure 3:**
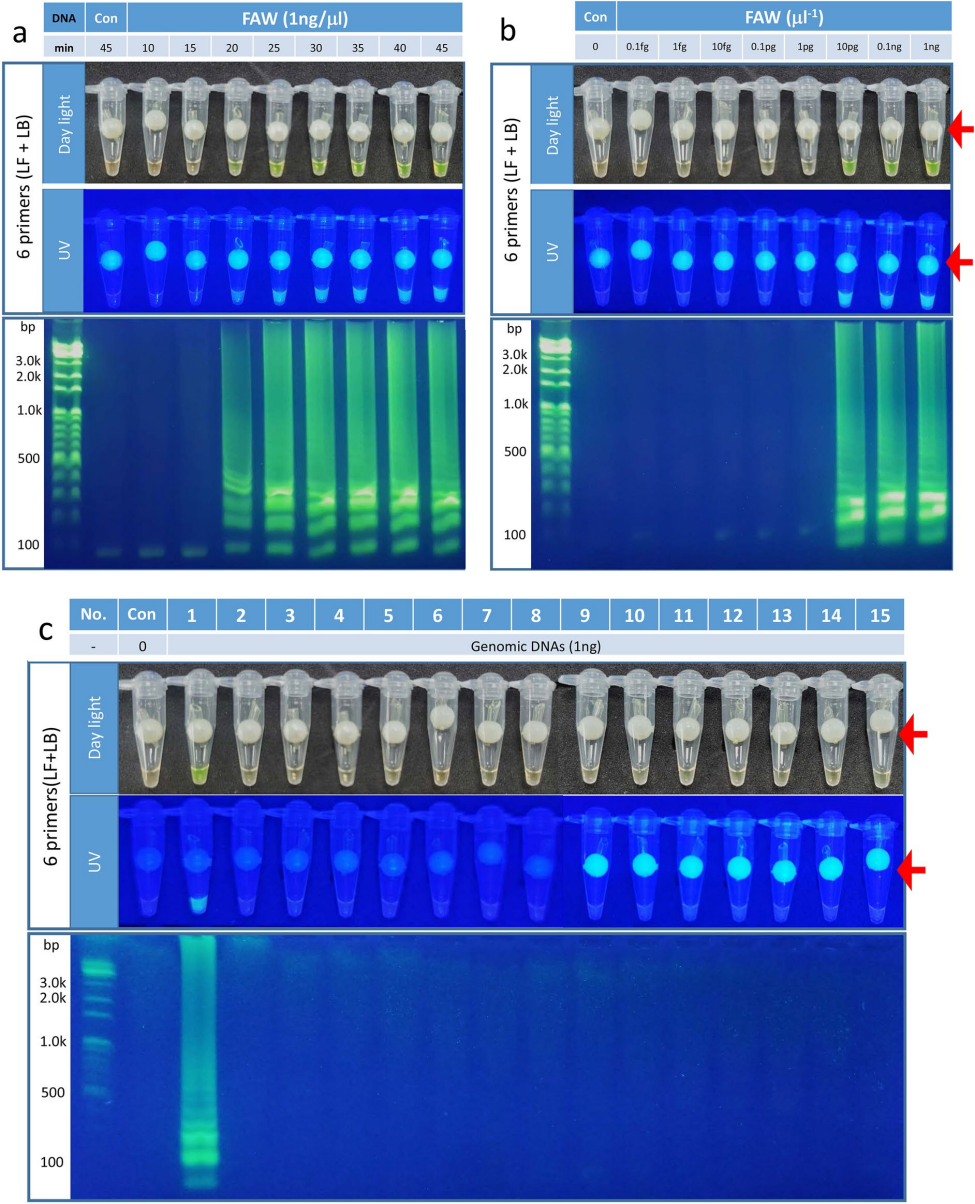
Improved efficiency of the Sf6p-LAMP assay with FAW species specificity. (**a**) After 20 min of incubation in Sf6p-LAMP assays, the fluorescence signals and amplification of DNA fragments were detected. In addition, the fluorescence signals and patterns of amplified DNA fragments were similar in Sf6p-LAMP assays after 25 min of incubation. (**b**) Since the fluorescence signal and DNA amplification were identified above 10 pg/μl, the detection limit for the Sf6p-LAMP assays was 10 pg/μl. (**c**) When 1 ng of genomic DNA from 15 moth species was used for Sf6p-LAMP assay, clear fluorescence signals and amplification of DNA fragments were detected from FAW genomic DNA only. Moth species used in this study were as follows: 1. FAW, 2*. Axylia putris*, 3*. Mythimna loreyi*, 4.* Discestra trifolii*, 5. *Hermonassa cecilia*, 6.* Anomis flava*, 7. *Aedia leucomelas*, 8.* Pleuroptya ruralis*, 9.* Mythimna separata*, 10. *Lacanobia contigua*, 11. *Helicoverpa armigera*, 12. *Mamestra brassicae*, 13*. Spodoptera exigua*, 14. *Spodoptera litura*, 15. *Plutella xylostella.* The red arrows indicate the contamination-free SYBR green delivery device.

Next, a Sf6p-LAMP assay was performed to investigate any change in incubation times. After 20 min of incubation, fluorescence signals and DNA amplifications were detected from Sf6p-LAMP assay. The fluorescence intensities and patterns of DNA fragments were similar after 25 min of incubation (Fig. 3a). These results suggested that the Sf6p-LAMP assay could reduce the incubation period by 10 min compared with that of the Sf4p-LAMP assay. In addition, similar fluorescence signals and patterns of DNA fragments of Sf6p-LAMP assay were detected when more than 10 pg/µl FAW genomic DNA was used (Fig. 3b). Furthermore, the Sf6p-LAMP assay only produced fluorescence signals and amplified DNA fragments when incubated with FAW genomic DNA, not with genomic DNA from other moths (Fig. 3c).

### The Sf6p-LAMP assay was able to detect FAW collected from Benin, Africa

**Figure 4:**
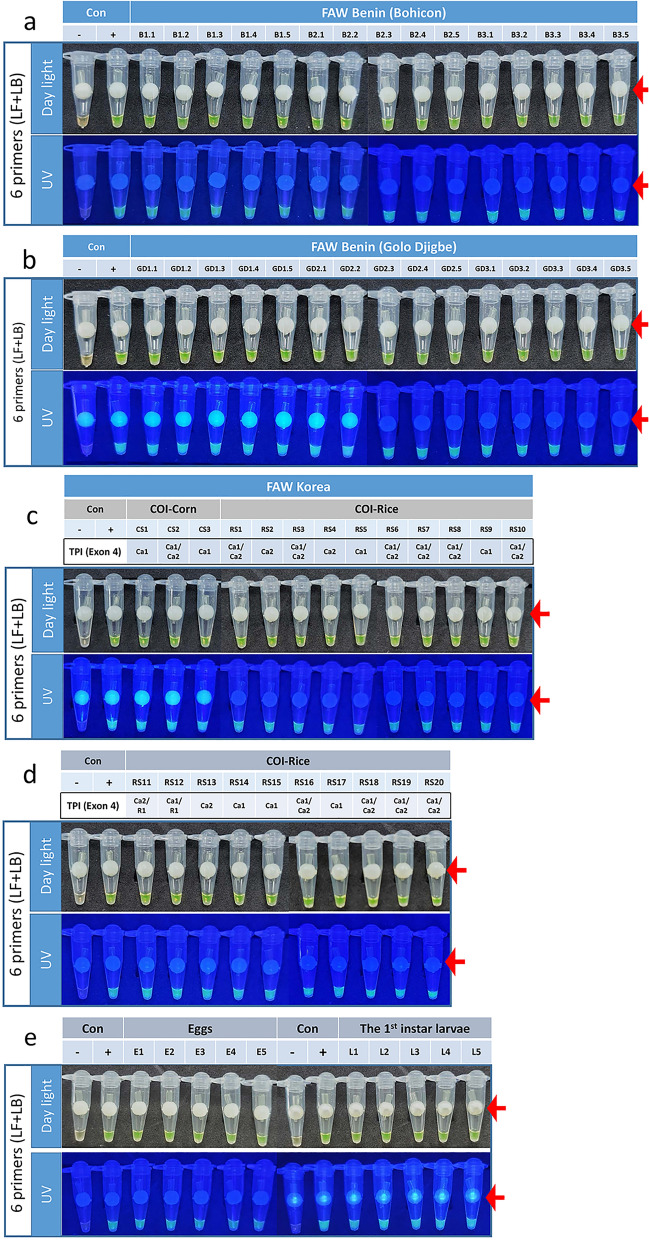
Sf6p-LAMP assays also recognized FAWs collected from Benin, Africa and eggs or 1st instar larvae of FAW within 30 min. (**a**) Sf6p-LAMP assays were performed on 15 FAW larvae collected from a corn field in Bohicon, Benin, Africa. (**b**) Fifteen FAW larvae collected in a corn field in Golo Djigbe were also tested with Sf6p-LAMP assays. (**c**, **d**) When 23 genomic DNAs from various genotype-based FAW strains were used for the Sf6p-LAMP assay, strong fluorescence signals were detected from all tested strains of FAW. (**e**) Eggs and 1st instar larvae of FAW were also detected by the Sf6p-LAMP assay. The red arrows indicate the contamination-free SYBR green delivery device.

Since FAWs invading Korea are considered to be FAW colonies that settled in China through Africa^15^, an Sf6p-LAMP assay specificity test was conducted using FAWs collected from two locations in Benin, Africa. When genomic DNA was extracted from a total of 30 FAW larvae collected from Bohicon and Golo Djigbe in Benin, a strong fluorescence signal was observed from all samples (Fig. 4a,b).

### The various *COI* and *Tpi*-based strains of FAW were detected by Sf6p-LAMP assays

FAWs are divided into rice and corn strains according to their feeding host. In a previous study investigating the COI sequences of FAW, we reported that most of the FAWs captured in FAW pheromone traps in Korea belonged to the rice strain^8^. However, recent studies have shown that the FAW found in Africa and Asia has complex origins and mutations different from that of the western hemisphere^16,17^. Therefore, in order to confirm the exact lineage of FAW, it is also necessary to study the genetic diversities of FAW using the DNA sequence of Triose phosphate isomerase (Tpi). We studied the genetic diversity of 23 FAWs collected from Korea by investigating the sequence of the 4th exon of *Tpi*. We found that all diagnostic markers of *Tpi* reported from African FAW were found in 23 Korean FAW samples. *Tpi* Ca1, Ca2, and Ra1 haplotypes were identified. Among the 23 FAW samples, one Ca1/Ra1—(4.3%), and one Ca2/Ra1—(4.3%), 7 Ca1—(30.4%), three Ca2—(8.7%), and 11 Ca1/Ca2-type samples (47.8%) were included (Fig. 4c,d). These genotyping results suggested that FAW collected in Korea may be descendant strains that migrated to China from Africa. Nevertheless, all of the Sf6p-LAMP assays using genomic DNA from 23 FAWs were positive (Fig. 4c,d), indicating that the Sf6p-LAMP assay could detect various *COI* and *Tpi*-based strains of FAW captured in Korea.

### Sf6p-LAMP assay could detect eggs and 1st instar larvae within 30 min

If invading FAW adults spawn eggs on crops, the damage to the crop can increase rapidly. Thus, protocols to diagnose eggs or 1st instar larvae of FAW are essential for proper control of this pest. Thus, we developed a diagnostic protocol that could be used for detecting eggs and 1st instar larvae of FAW in the field. When an egg or a 1st instar larva of FAW was ground for 2 to 3 min in a Quick DNA extraction solution, immediately mixed with Sf6p-LAMP mixture without further treatment, and then incubated for 25 min, positive fluorescence signals were observed in all samples (Fig. 4e). This result suggested that samples such as eggs or 1st instar larvae, which cannot not be morphologically classified, could be diagnosed on-site within 30 min.

### No false-positive or -negative reaction of the Sf6p-LAMP assay and the FAW diagnostic protocol in the validation experiments

To investigate the probability of false-positive or -negative reaction of the Sf6p-LAMP assay, we performed the Sf6p-LAMP assay using 10 ng of genomic DNA of 15 moths including FAW. A strong fluorescence signal was observed only from FAW tubes (Supplementary Fig. 2). In addition, FAW diagnostics were performed using genomic DNAs extracted from 50 FAW eggs and 50 *S. litura* eggs using a Quick DNA extraction solution (Supplementary Fig. 3). There were no false-positive or -negative reactions from 100 genomic DNAs from FAW and *S. litura* eggs (Table 1). These results suggested that the Sf6p-LAMP assay and the FAW diagnostic protocol can detect FAW very accurately.

**Table 1:** Summary of validation experiments for the Sf6p-LAMP assay and the FAW diagnostic protocol.

Experiments	The Sf6p-LAMP assay	The FAW diagnostic protocol
Samples	10 ng genomic DNA of 15 moths (triplicate)	100 genomic DNAs from 50 FAW eggs and 50 *S. litura* eggs
Positive/false-negative	3 FAW/not-detected	50 FAW eggs/not-detected
Negative/false-positive	14 moths × 3/not-detected	50 *S. litura* eggs/not-detected

## Discussion

Currently, the most obvious example of the damage caused by FAW's excellent migration ability is the situation in Korea. In Korea, FAW cannot winter because the average winter temperature is below 10 °C throughout the country. However, 6 months after FAW invasion into the tropical and subtropical regions of Southeast Asia was confirmed in early 2019, adult FAWs were trapped in the southern part of Korea. In 2020, adult FAWs were collected by pheromone traps in May, 1 month earlier than the previous year^8^. These reports suggest that the FAW, which has settled in Southeast Asia, continues to migrate to Korean peninsula. Therefore, the most important contribution of this study is the discovery of a FAW species-specific gene (Fig. 1) and establishment of a LAMP assay using this gene to accurately and quickly diagnose FAW (Figs. 2, 3, 4). While many previously developed LAMP assays for species determination usually use sequence differences within mitochondrial genomes^18^, we selected a FAW-specific gene using available RNA-seq datasets and then confirmed its specificity by comparison with 9 other moth species captured in FAW pheromone traps and 5 closely related moth species (Fig. 1). In addition, by examining various LAMP assays, the Sf6p-LAMP assay was found to have the highest sensitivity and accuracy (Figs. 2, 3, 4, Table 1).

Various diagnostic methods have been developed for species diagnosis, of which the most sensitive and highly accurate method is molecular diagnosis^19^. However, molecular diagnostics have several disadvantages that are not suitable for field applications. One of the common disadvantages of molecular diagnostics is the requirement of a sophisticated PCR machine^13,14^. This disadvantage can be solved by the development of a LAMP assay, since LAMP can be performed with only a simple thermostat^13,14,18,19^. Another disadvantage is that it takes a long time to extract genomic DNA from samples. In this study, this disadvantage was solved by using a Quick DNA extraction solution. When the genomic DNA extracted with Quick DNA extraction solution within 3 min was directly mixed with the Sf6p-LAMP mixtures and incubated for 25 min at 65 °C, FAW could be immediately confirmed by the presence of fluorescence signals in the tubes (Figs. 3, 4). In addition, we also confirmed the accuracy of the Sf6p-LAMP assays by performing the triplicate Sf6p-LAMP assays with genomic DNAs from 15 moths (Supplementary Fig. 2 and Table 1). Furthermore, the accuracy of the FAW diagnostic protocol were also confirmed by performing experiments with genomic DNAs from 50 FAW eggs and 50 *S. litura* eggs extracted with Quick DNA extraction solution (Supplementary Fig. 3 and Table 1). Therefore, the FAW diagnostic protocol that we have established, which has solved various problems of molecular diagnostic methods, is the second major contribution of this study. Recently, various LAMP-techniques have been developed to further improve the accuracy and sensitivity of LAMP technique and to solve the problem of contamination. Therefore, if our Sf6p-LAMP assay developed in this study were modified using the recently developed technologies^10–12^, it will be possible to develop a more accurate and faster diagnostic method in the near future.

Another contribution of this study is that our Sf6p-LAMP assay can diagnose FAWs captured in Africa and various *COI* and *Tpi*-based strains of FAWs trapped in Korea (Fig. 4). The reason this result is important is that FAWs are known to accumulate genetic mutations in their mitochondrial genomes as they continue to migrate and thus settle in new regions^8,20^. Therefore, it is a very important contribution to develop a diagnostic method using a structural gene that is less likely to have mutations than mitochondrial genes, in which mutations occur frequently and can have intraspecies mutations within a short period of time.

In summary, the FAW diagnostic protocol established in this study was completed from DNA extraction to diagnosis within a total of 30 min. Thus, this protocol can be easily and widely used to diagnose FAWs in various circumstances.

## Methods

### Identification of a fall armyworm specific gene

A total of 8 RNA-seq datasets of FAW were obtained from NCBI (https://www.ncbi.nlm.nih.gov/nuccore/GESP00000000.1, SRR3406020, SRR3406031, SRR3406036, SRR3406052, SRR3406053, SRR3406054, SRR3406055, and SRR3406059). Since SRR346059 had a defect, the 7 remaining RNA-seq datasets were used to generate 22,745 contigs (data not shown) by using the Trinity program^21^. To identify FAW-specific genes, 22,745 contigs were searched by BLASTX (https://blast.ncbi.nlm.nih.gov/Blast.cgi?LINK_LOC=blasthome&PAGE_TYPE=BlastSearch&PROGRAM=blastx) using Lepidopteran protein Refseq, which contains 387,000 proteins. The Lepidoptera protein Refseq was obtained from GenBank (https://www.ncbi.nlm.nih.gov/genbank/). The species specificity of Sf00067 (GenBank accession no: MW916664) was confirmed by performing conventional PCR using genomic DNA from various moths as described below.

### Collecting insects

FAW larvae were collected at a corn experimental station located in the Crop Cultivation and Environment Research Division, National Institute of Crop Science, Rural Development Administration (RDA), Suwon, Gyeonggi-do, Republic of Korea, and raised at the insectarium with a modified artificial diet [23 g agar, 3.6 g ascorbic acid, 1.8 g sorbic acid, 3 g methyl-p-hydroxybenzoate (DaeJeong Chemical & Metal Co., Siheung, Korea), 1400 ml distilled water, 75 g pinto bean powder, 60 g wheat germ, 30 g soybean meal (Bio-Serv, Flemington, NJ, USA), 30 g dried whole milk (Seoul Milk, Seoul, Korea), 37.5 g yeast (Ottogi Corporation, Anyang, Korea), and 5 g vitamin mixture (Seoul Vet Pharma Co. LTD, Seoul, Korea)]^22,23^.

Adult moths from 10 species, FAW, *Axylia putris*, *Mythiman lorevi*, *Discestra trifolii*, *Hermonassa cecilia*, *Anomis flava*, *Aedia leucomelas*, *Pleuroptya ruralis*, *Mythimna separata*, and *Lacanobia contigua,* trapped by FAW pheromone traps located at a corn experimental station, Nation Institute of Agricultural Sciences (NAS), RDA, Wanju, Jeollabuk-do, Korea, were used for extracting genomic DNA. Species of moths were confirmed by sequencing cytochrome oxidase I (COI). Larvae of *Helicoverpa armigera*, *Mamestra brassicae*, *Spodoptera exigua*, *Spodoptera litura*, and *Plutella xylostella* were raised at the insectarium at NAS, RDA, Wanju, Jeollabuk-do, Korea.

FAW larvae were collected from corn fields located in Bohicon and Golo Djigbe, Benin, Africa and stored in 70% ethanol for preservation.

### Extracting genomic DNA from insects

A Qiagen DNeasy Blood & Tissue genomic DNA extraction kit (Qiagen, Hilden, Germany) and its protocol were used to extract genomic DNA from samples. Briefly, approximately 100 mg of tissues was excised from samples and then ground with lysis buffer. After incubation for 2 h with proteinase K, large tissue debris was removed by centrifugation. Genomic DNAs were purified by using a DNA binding column and then eluted with dH_2_O. The concentrations of genomic DNA were measured by an Epoch microplate reader (BioTek, Winooski, VT, USA) and then diluted to 10 ng/μl. Other concentrations of genomic DNA were generated by diluting 10 ng/μl genomic DNA solutions with distilled water (DW). In addition, previously published genomic DNAs of 20 rice and 3 corn strains of FAW^8^ were used.

### FAW-specific primer sets for Sf00067 loop-mediated isothermal amplification (LAMP) and universal COI primer pairs

Six primers for performing Sf00067-LAMP were designed as previously published^13,14^. The DNA sequences of the 6 primers are listed in Supplementary Table 1. The previously published universal COI primers LCO1490 and HCO2198^24^ were used as positive PCR controls to verify the quality of the extracted genomic DNA (Supplementary Table 1).

### A conventional PCR protocol

The specificity of Sf00067 was verified by performing conventional PCR using the Sf00067 F3 and B3 primer pairs (Supplementary Table 1). PCR mixtures contained 0.2 μM F3 and B3 each, 1 × rTaq master mix (iNtRON Biotechnology, Seong-nam, Korea), and 1.0 ng/μl of genomic DNA. The PCR program was 3 min of incubation at 95 °C and then 35 cycles of denaturing at 95 °C for 10 s, annealing at 60 °C for 10 s and extension at 72 °C for 10 s. PCR was terminated after a 10 min extension at 72 °C, and then products were separated with 1.5% agarose gel (1X TBE buffer) electrophoresis containing 1 × NEOgreen (Cellgentek, Cheongju, Korea). To verify that the quality of genomic DNA extracted from various moths was sufficient for PCR, a previously published universal COI primer pair was used^24^ (Supplementary Table 1). The PCR mixture and conditions were the same as above except that the annealing temperature was 50 °C. The sequences of both strands of PCR products were confirmed by capillary sequencing (data not shown).

### LAMP protocols

Previously published LAMP assay protocols^13,14^ were modified as outlined below. The Sf4p-LAMP assay mixture contained 1 × I buffer 2, 0.15 units of GspSSD polymerase/μl, 1.6 M betaine, 10 mM MgSO_4_ (Optigene Limited, Horsham, England), 1.6 mM dNTPs (Enzynomics, DaeJeon, Korea), 0.2 M trehalose (Merck KGaA, Darmstadt, Germany), 0.2 µM F3, 0.2 µM B3, 0.8 µM forward internal primer (FIP), 0.8 µM reverse internal primer (BIP) and 1.0 ng/μl genomic DNA and was incubated at 65 °C for 15 to 50 min. SYBRgreen (Thermo-Fisher Scientific, Waltham, MA, USA) loaded in a contamination-free dye delivery device for LAMP assays (Primer4Dia, Anyang, Korea) was added after incubation at 65 °C by quick spinning with a portable microcentrifuge (DaiHan Sci., Seoul, Korea).

The SfLF-LAMP assay was performed by adding 0.4 µM LF to the Sf4p-LAMP assay mixture followed by incubation at 65 °C for 15–50 min. Similarly, the SF00067 loop reverse primer (SfLB)-LAMP assay was performed by adding 0.4 µM LB to the Sf4p-LAMP assay mixture followed by incubation at 65 °C for 15–50 min. The Sf6p-LAMP assay was performed by adding 0.4 µM LF and 0.4 µM LB to the Sf4p-LAMP assay mixture with incubation at 65 °C for 10–45 min.

To determine the detection limits for the Sf00067-LAMP assays, 1 fg to 10 ng of serially diluted FAW genomic DNA was added to various Sf00067-LAMP assays described above. Incubation periods at 65 °C for Sf4p- or SfLB-LAMP assays were 35 min and 25 min for the SfLF- or Sf6p-LAMP assays, respectively.

The results of the Sf00067-LAMP assays were initially determined by fluorescence signals under daylight and UV light. Amplification of DNA fragments by LAMP assays was determined by 1.5% agarose gel electrophoresis (1 × TBE).

### FAW Triose phosphate isomerase (*Tpi*) genotyping protocol

Genotypes of 23 FAW samples collected in Korea were identified by performing *Tpi* genotyping protocol published by Nagoshi et al.^16^. Briefly, the fourth exon of TPI DNA fragments were amplified using Tpi-412 forward (5′-CCGGACTGAAGGTTATCGCTTG-3′) and Tpi-850 reverse (5′-AATTTTATTACCTGCTGTGG-3′) primers. PCR amplification was performed in a 20-µl reaction mixture containing 4 µl of r-Taq Plus Hot 5X Master Mix (Eplis biotech. Daejeon, Korea) and 2 µl of genomic DNAs (10–100 ng). The PCR amplification was performed using an ABI 2720 thermal cycler (Thermo Fisher Scientific), with the thermocycling program consisted of 95 °C (7 min), followed by 35 cycles of 95 °C (10 s), 56 °C (10 s), 72 °C (10 s), and a final incubation of 72 °C for 10 min. Amplified DNA fragments were mixed with a 5 µl of 5 × gel loading buffer and then separated by 2.0% agarose gel containing 1 × Safe-Pinky (GenDEPOT, Katy, TX, USA). DNA fragments visualized by an UV-transilluminator (DaeHan Sci, Seoul, Korea) were excised with a razor blade and then purified with Nucleospin Gel and PCR clean up kit (Takara Bio., Shiga, Japan) as manufacturer’s protocols. The DNA sequencing of both stranded of DNAs was performed at COSMOgenetech (Seoul, Korea). DNA sequence alignments and analyses were performed using CLC Sequence Viewer 8.0 (Qiagen Digital Insight, Arthus, Denmark).

### FAW diagnostic protocol and validation experiments

An egg or a 1st instar larva of FAW was ground with 30 or 200 μl of Quick DNA extraction solution (Primer4Dia), respectively, for three minutes for genomic DNA extraction. After mixing the genomic DNA extracts and Sf6p-LAMP mixtures at a ratio of 1:20, the mixture was incubated for 25 min at 65 °C. The species was determined based on the presence or absence of fluorescence in tubes under day light and UV light. The total duration for the FAW diagnostic protocol was approximately 30 min.

For validating the accuracy of Sf6p-LAMP assays, 10 ng of genomic DNAs from 15 moths including FAW were performed. A triplicate experiment was completed. In addition, for validating the accuracy of the FAW diagnostic protocol described above, genomic DNAs extracted from 50 FAW eggs and 50 *S. litura* eggs with Quick DNA extraction solution (Primer4Dia) were used to perform. The negative (no genomic DNA) and positive controls (1 ng of FAW genomic DNA) were prepared every time.

## Supplementary Information

Supplementary Information.

## Data Availability

The datasets used in this study are available from the corresponding author on reasonable request.
