# Supplementary material for: Identification of a fall armyworm (Spodoptera frugiperda)-specific gene and development of a rapid and sensitive loop-mediated isothermal amplification assay
Source: Sci Rep. 2022 Jan 18;12:874. doi: 10.1038/s41598-022-04871-2 (PMC8766445; doi:10.1038/s41598-022-04871-2)
Supplement: Supplementary file 1 — Supplementary Information. [file 41598_2022_4871_MOESM1_ESM.pdf]

Identification of a fall armyworm (*Spodoptera frugiperda*)-specific gene and development of a rapid and sensitive loop-mediated isothermal amplification assay

Angelina F. Osabutey[1,2*], Bo Yoon Seo[3*], A-Young Kim[1,2*], Thu Ahn Thi Ha[1,2*], Jin-Kyo Jung[4], George Goergen[5], Ebenezer Oduro Owusu[6], Gwan-Seok Lee[3] and Young Ho Koh[1,2P]

[1] Department of Biomedical Gerontology, Hallym University Graduate School, Chuncheon, Gangwon-Do, Republic of Korea

[2] Ilsong Institute of Life Science, Hallym University, Yeongdeungpo-gu, Seoul, Republic of Korea

[3] Crop Cultivation and Environment Research Division, National Institute of Agricultural Sciences, Rural Development Administration, Wanju, Jeollabuk-Do, Republic of Korea

[4] Central Crop Division, National Institute of Crop Sciences, Suwon, Gyeonggi-do, Republic of Korea

[5] IITA Biological Control, Center for Africa, 08BP 0932, Tri postal, Cotonou, Benin

[6] Department of Animal Biology and Conservation science, University of Ghana, Legon-Accra, Ghana

* These authors contributed equally.

Short Title: Rapid and sensitive FAW diagnosis with LAMP

P Correspondence, Young Ho Koh, Ph.D. email: Kohyh@hallym.ac.kr, Ilsong Insitute of Life Sciences, Hallym University, Seoul. Korea Tel: 82-10-9979-3703

Supplementary Table 1. Oligonucleotide sequences of 6 primers designed for Sf00067 and universal COI primers

Name	Sequences
Sf00067 Forward primer 3 (F3)	CCAAGATACGTAGTTATTGGTT
Sf00067 Reverse primer 3 (B3)	CCATAATAAGACTGTTGAAATCGA
Sf00067 Forward internal primer (FIP)	CTGACGTCACGAATATAACAGTGATCAAATAGAAAAAACAATGCAGGT
Sf00067 Reverse internal primer (BIP)	TCCGTATGAAAATCTAAACGCTAGTGTGTATTGCTCGTATATTAATGC
Sf00067 Loop forward primer (LF)	CGAACTGGGACATGTCAGA
Sf00067 Loop reverse primer (LB)	TTTTCAAATAACAAAATT
LCO1490[24]	GGT CAA CAA ATC ATA AAG ATA TTG G
HCO2198[24]	TAA ACT TCA GGG TGA CCA AAA AAT CA

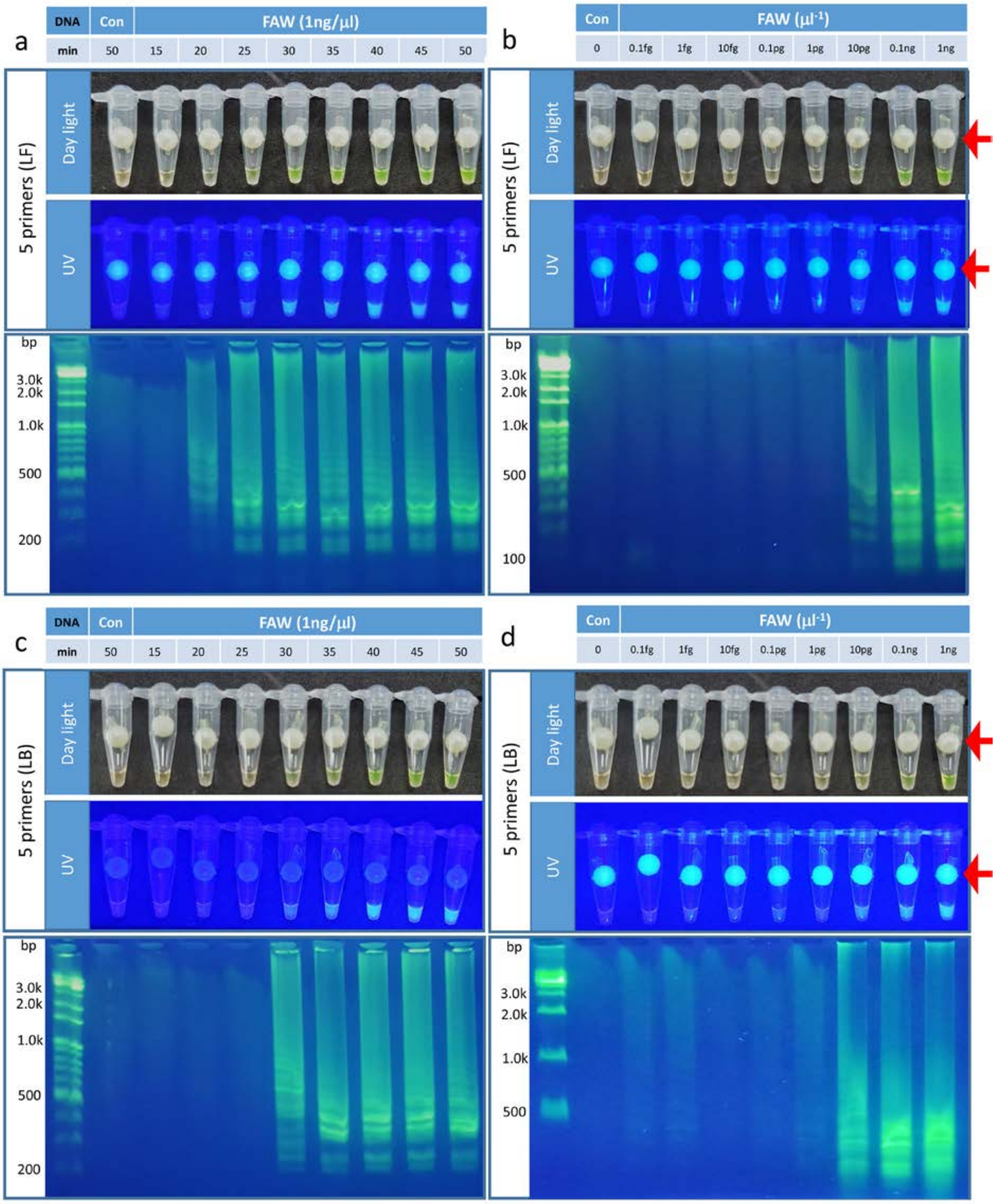

Supplementary Figure 1. The efficiency of the Sf4p-LAMP assay was enhanced by supplementing loop primers.

A. The SfLP-LAMP assay showed a reduced incubation time by 10 minutes compared to the Sf4p-LAMP assay. B. The detection limit of the SfLF-LAMP assay was 10 pg/μl, since fluorescence signals and DNA amplification were detected when 10 pg/μl of FAW genomic DNA was used. C. The SfLB-LAMP assay did not show a reduced incubation time compared to that of the Sf4p-LAMP assay. D. The detection limit of the SfLB-LAMP assay was 10 pg/μl, 10 times higher than that of the Sf4p-LAMP assay. The red arrows indicate the contamination-free SYBR green delivery device.

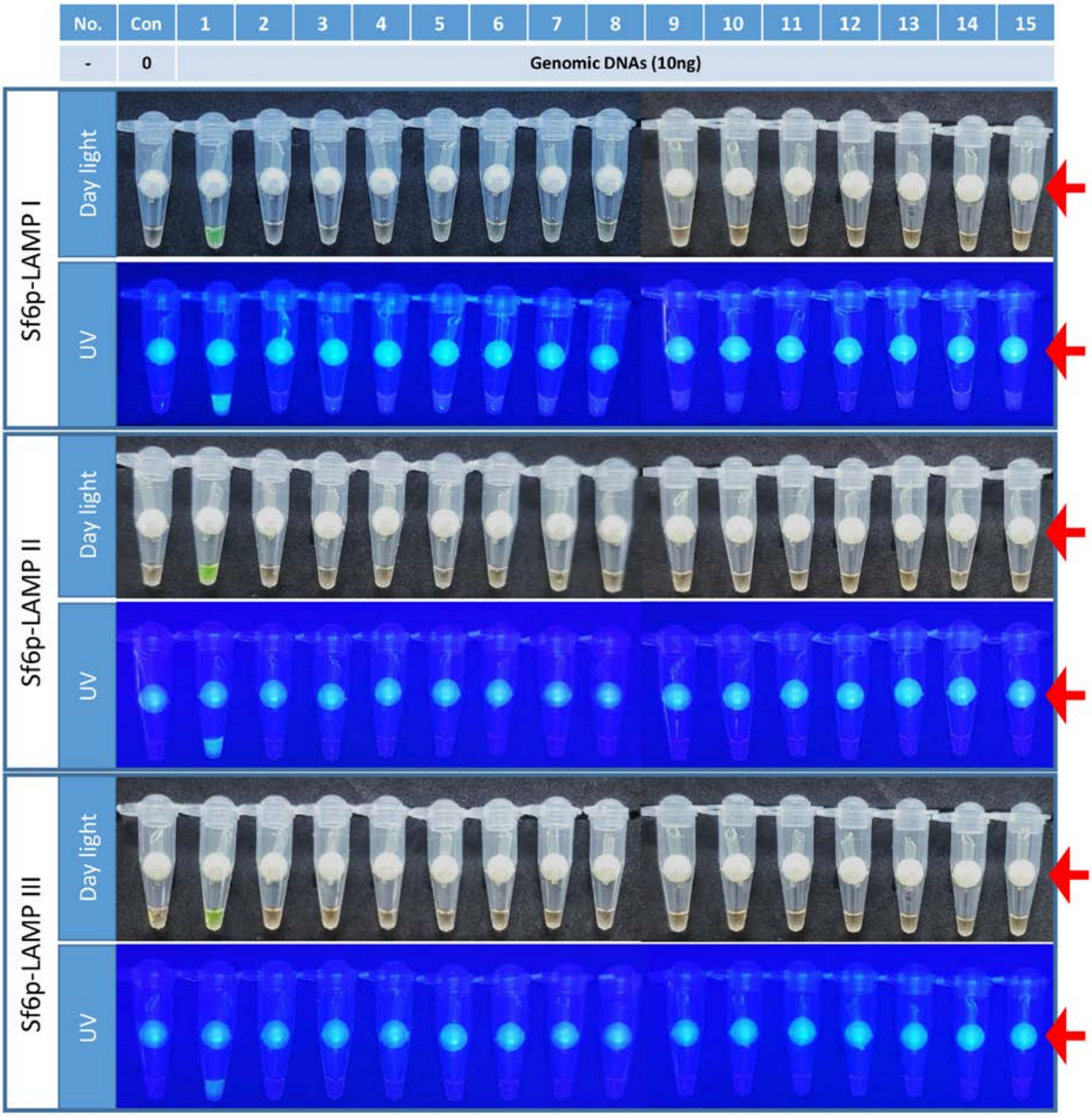

Supplementary Figure 2. Accuracy verification test results of the Sf6p-LAMP assay for 15 different moth species

When 10 ng of genomic DNAs from 15 moths were used for Sf6p-LAMP assays, strong fluorescence signals were only detected from a tube containing FAW

genomic DNAs. Three replications were performed. The red arrows indicate the contamination-free SYBR green delivery device. Moth species used in this study were as follows: 1. FAW, 2*. Axylia putris,* 3*. Mythimna loreyi, 4. Discestra trifolii,* 5. *Hermonassa cecilia,* 6*. Anomis flava,* 7. *Aedia leucomelas,* 8*. Pleuroptya ruralis,* 9*. Mythimna separata,* 10. *Lacanobia contigua,* 11. *Helicoverpa armigera,* 12. *Mamestra brassicae*, 13*. Spodoptera exigua,* 14. *Spodoptera litura,* 15. *Plutella xylostella.*

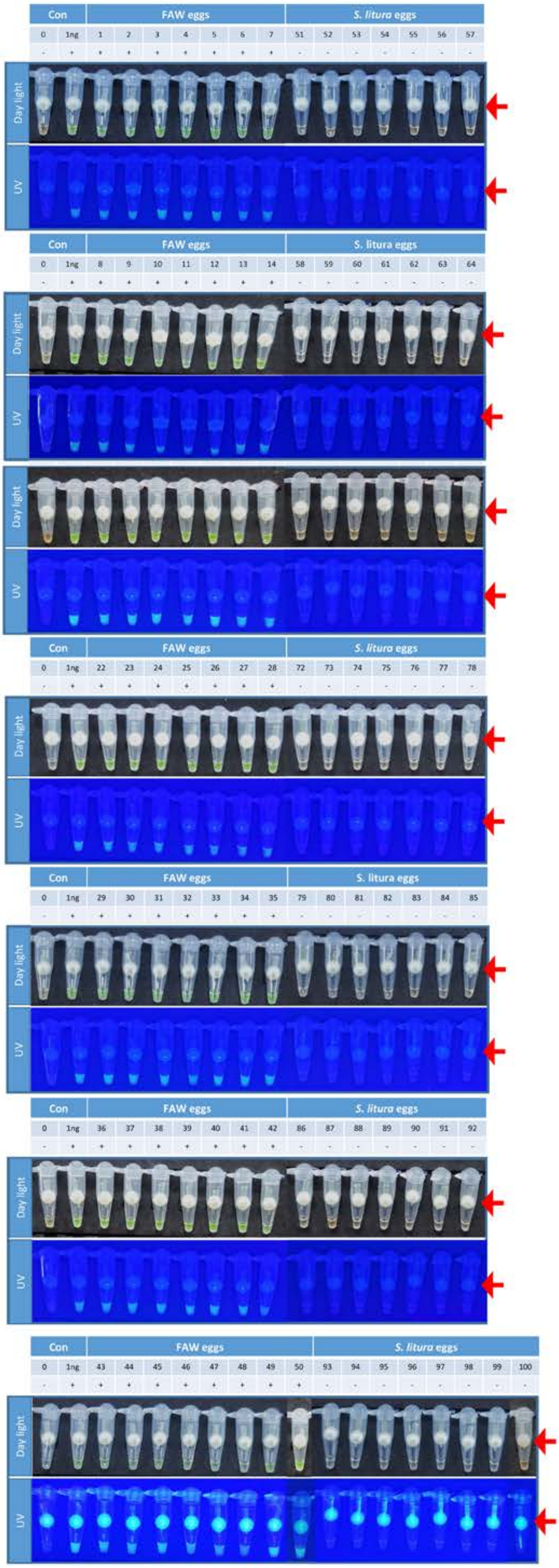

Con
FAW eggs
S. litura eggs
0 1ng 1 2 3 4 5 6 7 51 52 53 54 55 56 57
0 1ng 8 9 10 11 12 13 14 58 59 60 61 62 63 64
0 1ng 22 23 24 25 26 27 28 72 73 74 75 76 77 78
0 1ng 29 30 31 32 33 34 35 79 80 81 82 83 84 85
0 1ng 36 37 38 39 40 41 42 86 87 88 89 90 91 92
0 1ng 43 44 45 46 47 48 49 50 93 94 95 96 97 98 99 100
Day light
UV

Supplementary Figure 3. The accuracy of the Sf6p-LAMP assay

When genomic DNAs from 50 FAW eggs and 50 *S. litura* eggs were used for the Sf6p-LAMP assays, strong fluorescence signals were detected from 50 genomic DNAs from FAW eggs. The red arrows indicate the contamination-free SYBR green delivery device.

References

24 Folmer, O., Black, M., Hoeh, W., Lutz, R. & Vrijenhoek, R. DNA primers for amplification of mitochondrial cytochrome c oxidase subunit I from diverse metazoan invertebrates. *Mol. Mar. Biol. Biotechnol.* **3**, 294-299 (1994).
